# Evaluation of bias and precision in methods of analysis for pragmatic trials with missing outcome data: a simulation study

**DOI:** 10.1186/1745-6215-14-S1-P110

**Published:** 2013-11-29

**Authors:** Royes Joseph, Julius Sim, Reuben Ogollah, Martyn Lewis

**Affiliations:** 1 Keele University, Staffordshire, UK

## 

Randomised controlled trials (RCTs) in arthritis and musculoskeletal conditions generally necessitate long-term follow up of largely self-reported outcomes; thus, such RCTs are prone to missing outcome data, mainly because of participant dropout/non-response. Recent years have seen a rise in the application of methods for dealing with missing outcome data (e.g. mixed models for repeated measures or multiple imputation). However, the implications of the missing data and their handling in pragmatic RCTs (as in arthritis and musculoskeletal conditions) have not received widespread attention to date. In a review of 91 published RCTs in arthritis and musculoskeletal conditions in 2010-11, we found that complete case analysis and single imputation – such as last observation carried forward – are still the most commonly used approaches to analysis of the primary endpoint. None of the RCTs reported a primary analysis or sensitivity analysis based on an assumed ‘missing not at random’ mechanism. The findings indicate a possible belief among researchers that if the dropout rate is low and/or equal between treatment arms, bias is not a concern and advanced methods to handle dropouts are unnecessary. In this study we perform a detailed simulation aimed at understanding the nature and degree of bias in estimates of treatment effect in terms of the level of dropout, the pattern of dropout, the analysis used, and the type of missing data mechanism.

